# Intraindividual crossover comparison of gadobenate dimeglumine-enhanced and gadoxetate disodium-enhanced MRI for characterizing focal liver lesions

**DOI:** 10.1186/s41747-025-00551-8

**Published:** 2025-02-18

**Authors:** Wenjie Tang, Yuanqiang Xiao, Sichi Kuang, Dailin Rong, Bingjun He, Luigi Grazioli, Shahid M. Hussain, Jin Wang

**Affiliations:** 1 Department of Radiology, Third Affiliated Hospital of Sun Yat-Sen University, Guangzhou, PR China; 2 Department of Radiology, University of Brescia, Ospedale “Spedali Civili”, Brescia, Italy; 3 Department of Radiology, Stony Brook University Hospital, Stony Brook, NY USA

**Keywords:** Contrast media, Gadobenic acid, Gadolinium ethoxybenzyl DTPA, Liver neoplasms, Magnetic resonance imaging

## Abstract

**Background:**

Gadobenate and gadoxetate are hepatobiliary magnetic resonance imaging (MRI) contrast agents. We intraindividually compared these two agents for the characterization of focal liver lesions (FLLs).

**Methods:**

A total of 140 adult subjects were randomized to undergo two 3-T MRI exams separated by 7–14 days, one with 0.05 mmol/kg gadobenate and one with 0.025 mmol/kg gadoxetate. For both exams, we acquired the same unenhanced T1-weighted, T2-weighted, and diffusion-weighted sequences, followed by contrast-enhanced T1-weighted sequences during the dynamic and hepatobiliary phases (HBP) (at 20 min for gadoxetate, at 120 min for gadobenate). Three experienced unaffiliated readers independently evaluated each exam in blinded, randomized order for lesion nature (benign/malignant) and specific lesion diagnosis. McNemar test, Wald tests. paired *t*-tests and κ statistics were used.

**Results:**

A total of 208 FLLs (108 malignant and 100 benign) were confirmed at final diagnosis. Sensitivity and specificity for malignant/benign differentiation ranged from 91.6% to 99.1% and from 87.5% to 90.5% for gadobenate, and from 86.0% to 91.6% and from 79.7% to 83.6% for gadoxetate. Significantly (*p* ≤ 0.025) higher values for gadobenate were determined for all diagnostic performance parameters except for sensitivity and negative predictive value for one reader. Significantly (*p* < 0.001) greater accuracy and confidence for specific lesion diagnosis was achieved with gadobenate for two of three blinded readers. Interreader agreement for malignant/benign differentiation was better with gadobenate (κ = 0.91 *versus* κ = 0.72).

**Conclusion:**

Gadobenate was superior to gadoxetate for the differentiation and diagnosis of malignant and benign FLLs for two of three readers. Further confirmatory studies that include a wider representation of different types of FLLs are warranted.

**Relevance statement:**

Better diagnostic performance and greater confidence in the characterization of FLLs with gadobenate might improve patient management decisions and timings, and potentially lead to better patient outcomes.

**Key Points:**

- Better diagnostic performance for the differentiation of FLLs was achieved with gadobenate for two of three readers.
- Reader confidence for lesion diagnosis was greater with gadobenate.
- Superior dynamic phase imaging with gadobenate was crucial for accurate lesion diagnosis.

**Graphical Abstract:**

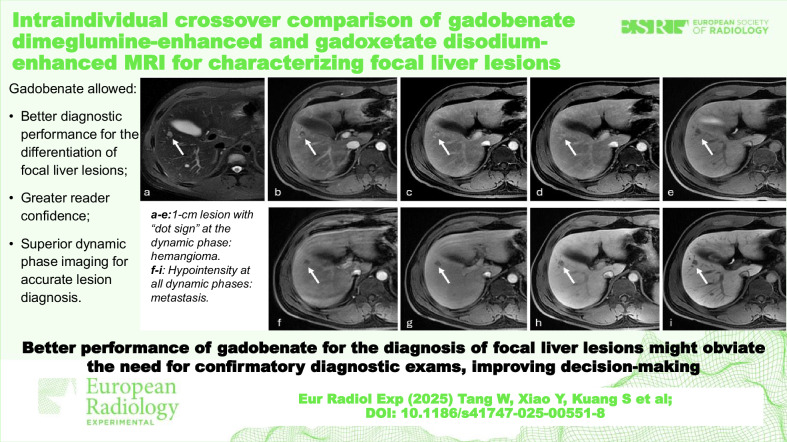

## Background

The fundamental aims of diagnostic liver imaging are to differentiate malignant from benign focal liver lesions (FLLs) and to establish their correct diagnosis. This allows treatment plans to be developed for patients with liver cancer while unnecessary additional diagnostic procedures (*e.g*., biopsy) can be avoided in patients with clearly benign or no neoplasms. Currently, magnetic resonance imaging (MRI) is considered the most reliable technique for diagnostic liver imaging due to its noninvasiveness, high intrinsic soft-tissue contrast, multiplanar capability, high level of anatomical and functional information, and lack of exposure to ionizing radiation [1].

Typically, gadolinium-based contrast agents (GBCAs) are used to improve the identification of FLLs and to increase the accuracy of their diagnosis. Of the GBCAs currently available, extracellular fluid GBCAs (gadoterate meglumine, Dotarem^®^, Guerbet; gadoteridol, ProHance^®^, Bracco; gadobutrol, Gadovist^®^, Bayer; and gadopiclenol, Vueway^®^, Bracco, Elucirem^®^, Guerbet) offer the opportunity to exploit differential vascular perfusion between normal liver parenchyma and FLLs thereby enabling characterization on imaging performed during the arterial, portal-venous, and equilibrium/delayed phases of contrast distribution. However, these GBCAs offer no benefit for FLL characterization beyond the initial dynamic phase of contrast distribution. On the other hand, hepatobiliary (HB)-GBCAs offer the possibility for FLL identification and characterization during both the postinjection dynamic phase of contrast distribution and during a delayed hepatobiliary phase (HBP), when FLLs without functioning hepatocytes invariably appear hypointense to the enhanced normal parenchyma on T1-weighted images due to their inability to take up these GBCAs [2, 3].

Two HB-GBCAs are commercially available: gadoxetate disodium (Primovist^®^/Eovist^®^, Bayer) and gadobenate dimeglumine (MultiHance^®^, Bracco). Whereas these agents both offer a dual imaging capability, gadoxetate is taken up more rapidly and to a quantitatively greater extent by functioning hepatocytes; approximately 50% of the injected dose of gadoxetate is taken up compared to 3–5% for gadobenate. A consequence of the more rapid uptake of gadoxetate is that HBP imaging is commonly performed 20 min after injection compared to 1–3 h for gadobenate [2]. However, a drawback of this more rapid uptake is that late dynamic phase sequences are often compromised by early intracellular uptake, often resulting in a pseudowashout appearance of hypervascular FLLs [4−6]. As a result, the dynamic phase of gadoxetate comprises an arterial phase, an early portal-venous phase, and a transitional phase (TP) beginning about 3 min after injection. Familiarity with the similarities, differences, and drawbacks of these agents is crucial for accurate FLL characterization [3–5].

Several studies have compared gadoxetate and gadobenate for liver imaging using an interindividual parallel-group study design and demonstrated benefits for one agent or the other depending on the patient and lesion populations evaluated [6–10]. Unfortunately, conclusions from parallel-group studies should be made with caution as these studies are unavoidably impacted by intergroup differences in patient and lesion populations. A more robust and reliable intraindividual crossover study design sees each patient undergo separate examinations with both agents such that any differences in diagnostic performance can be ascribed solely to differences in GBCA efficacy, if all remaining conditions are kept unchanged. Although several intraindividual crossover studies have compared gadoxetate and gadobenate for FLL characterization [11–15], these have typically included too few patients to confirm potential differences with adequate statistical power. On the other hand, a recent large-scale intraindividual crossover study in patients with suspected hepatocellular carcinoma (HCC) revealed highly significant superiority for gadobenate for the accurate diagnosis of “Liver imaging reporting and data system” (LI-RADS) 5 lesions [16].

Considering the paucity of adequately powered, large-scale intraindividual crossover studies, we prospectively compared gadoxetate and gadobenate with sufficient statistical power to potentially identify differences between the two agents for FLL characterization.

## Methods

### Patients

This was a single-center, prospective, intraindividual, randomized, double-blind comparison of gadobenate and gadoxetate for FLL diagnosis. Male and female subjects of at least 18 years were eligible for inclusion if they were referred for liver MRI for the investigation of untreated known or suspected FLLs or for evaluation of chronic liver disease. Eligible patients typically had lesions detected incidentally on ultrasound or computed tomography, had compensated cirrhosis and were undergoing surveillance, or were referred by gastroenterologists/oncologists for histologically confirmed colorectal cancer. Patients were ineligible for inclusion if they were contraindicated to GBCAs or MRI or were expected to receive any other diagnostic examination or treatment between the two GBCA administrations. Patients were also ineligible if they were pregnant or nursing or had impaired renal function (estimated glomerular filtration rate < 30 mL/min).

Histological confirmation was obtained for all malignant FLLs while histological confirmation or 12-month imaging follow-up confirmation was obtained for benign lesions.

Patients were prospectively randomized to two study groups (groups A and B). Patients in group A received gadobenate for the first examination and gadoxetate for the second examination. Patients in group B received the agents in the opposite order.

### MRI protocol

All examinations were performed using a 3-T scanner (Discovery MR750, GE Healthcare, Chicago, US) equipped with an eight-channel phased-array body coil. Patients were examined in the supine position with their arms alongside their bodies. The MRI protocol and scanning parameters were identical for all patients for both GBCAs.

Baseline MRI sequences included: (i) an axial respiratory-triggered single-shot echo-planar diffusion-weighted sequence with *b* values of 10 mm^2^/s and 800 mm^2^/s; (ii) an axial fat-suppressed three-dimensional T1-weighted spoiled gradient-echo with volume acceleration-flexible−LAVA-Flex sequence acquired in-phase and out-of-phase; and an axial respiratory-triggered fat-saturated T2-weighted fast spin-echo sequence. Dynamic imaging was performed in the axial plane using a three-dimensional T1-weighted volume acceleration-flexible sequence acquired during the early and late arterial phase at 15–25 s and 25–30 s, respectively, the early and late portal-venous phase at 45–60 s and 60–75 s, respectively, and at 2 min, 3 min, 5 min, and 10 min, termed “TP”, for gadoxetate and “delayed phase” for gadobenate. T1-weighted volume acceleration-flexible HBP images were acquired at 20 min for gadoxetate or at 120 min for gadobenate. Acquisition of two arterial (early and late) and two portal-venous (early and late) phase image sets was performed to preclude or reduce the possibility of nondiagnostic images associated with motion- or respiratory-related artifacts.

Gadoxetate and gadobenate were administered intravenously using a power injector at a rate of 2 mL/s as a single dose of 0.025 mmol/kg (0.1 mL/kg) and 0.05 mmol/kg (0.1 mL/kg), respectively, followed by 20–30 mL of saline flush at the same rate. The interval between the two MRI examinations was in all cases between 7 days and 14 days.

### Image interpretation

Images were anonymized and interpreted independently by three board-certified radiologists (L.G. and S.M.H., with 35 years and 24 years of post-training experience in liver imaging, respectively, and a non-author with 17 years of post-training experience in liver imaging) who had familiarity with the two HB-GBCAs and who were unaffiliated with the study center and blinded to all examination and patient information including clinical and histopathological data. Each reader received dedicated training on how to perform the reading. Each reader independently evaluated each MRI exam of all patients in randomized order.

To minimize recall bias, images were read in two distinct sessions separated by at least two weeks. During the first session either the images with gadobenate or the images with gadoxetate from each patient were evaluated. In the second session, the images from the other examination were evaluated. All images including unenhanced and contrast-enhanced scans (comprising both dynamic and HBP images) were evaluated together as a single exam for each agent.

#### Lesion detection

Each reader evaluated all available images for each patient and identified and mapped lesions based on the Couinaud classification of liver anatomy [17]. Lesion size (lesion maximum diameter in mm) was recorded. Readers were instructed to disregard simple cysts in their evaluations.

#### Lesion characterization

Lesion features on pre-contrast sequences were assessed in terms of: (i) lesion signal intensity on T1-weighted in-phase and out-of-phase sequences relative to the normal liver (1 = hypointense; 2 = isointense; 3 = hyperintense; and 4 = not seen); (ii) lesion signal intensity on T2-weighted sequences (1 = hypointense; 2 = isointense; 3 = mild-to-moderate hyperintensity; and 4 = marked hyperintensity); (iii) evaluation of diffusion-weighted images (1 = restricted diffusion, *i.e*., intensity not attributable solely to T2 shine-through, unequivocally higher than that of normal liver and/or apparent diffusion coefficient unequivocally lower than that of the normal liver; 2 = no restricted diffusion; 3 = T2 shine-through; and 4 = targetoid restriction) and it’s signal intensity (1 = hypointense; 2 = isointense; 3 = hyperintense; and 4 = not seen]); (iv) infiltrative appearance (yes or no); and (v) capsule presence (yes or no). Lesion features on contrast-enhanced dynamic images, including arterial, portal-venous, and TP/delayed phase images, were evaluated in terms of the pattern of enhancement using criteria for FLL diagnosis from the American Association for the Study of Liver Disease (AASLD) [1]. Based on these criteria, patterns of enhancement were classified as typical or atypical for each individual FLL and contrast agent. Lesions in the HBP were assessed in terms of signal enhancement using a 4-point scale (1 = hypointensity; 2 = isointensity; 3 = hyperintensity; 4 =  targetoid appearance).

#### Lesion diagnosis

Diagnosis of lesions as benign or malignant, as well as designation of a specific diagnosis, was based on their appearance on unenhanced (T1-, T2-, and diffusion-weighted) and contrast-enhanced (dynamic and HBP) images relative to the established features of FLLs described elsewhere [1, 18–22]. Each reader first determined if the detected lesions were malignant or benign. Thereafter, they assigned either a single specific diagnosis or differential diagnoses. A single specific diagnosis was assigned from a predefined list of diagnoses that included HCC, cholangiocellular carcinoma (CC), metastasis, or ‘other’ for malignant lesions, and hemangioma, focal nodular hyperplasia, hepatic adenoma, dysplastic nodule (DN), regenerating nodule, focal fatty infiltration, peliosis hepatis, abscess, or ‘other’ for benign lesions. A maximum of three differential diagnoses were permitted when readers chose not to assign a single specific diagnosis. When differential diagnoses were assigned, the first differential diagnosis was that which the reader was most confident about while the second or third differential diagnosis was that which the reader was less or least confident about.

Once all independent blinded readings were completed and locked, tracking of detected lesions to lesions detected at final diagnosis (either histopathology or imaging follow-up) was performed.

### Reference standard

Histological assessment of surgical and/or biopsy samples was performed for all lesions considered malignant by onsite investigators following the two MRI examinations. The final diagnosis of lesions considered benign was based on follow-up findings from MRI, computed tomography, and/or ultrasound assessed by an experienced radiologist not involved in the analysis of study images, as well as on clinical history and laboratory findings. Criteria for the diagnosis of lesion benignity at imaging follow-up consisted of stability in the number, size, and appearance of lesions over a period of 12 months. Clinical follow-up involved close medical observation of the patient for any clinical or laboratory signs of malignant disease over the 12-month period. The lack of any clinical or laboratory signs of malignant disease was considered indicative of lesion benignity.

### Statistical analysis

Assessments of diagnostic performance for lesion characterization were performed for each reader separately for lesions detected in each of the two MRI examinations. Calculation of sensitivity, specificity, accuracy, and positive and negative predictive values for the differentiation of benign and malignant FLLs was performed at the lesion level with final diagnosis results taken as reference standard. Differences in sensitivity, specificity, and accuracy between gadobenate and gadoxetate for the characterization of benign and malignant FLLs were assessed using the McNemar test. Differences in predictive values were tested using the Wald test from the generalized estimating equation (GEE) model with adjustment for within-patient correlation due to repeated measurements within a patient. The agreement between MRI and histology results for the determination of specific diagnoses was estimated for each HB-GBCA with their 95% confidence interval and compared using the McNemar test. A significance level (α error) of 0.05 was applied to all statistical tests.

Inter-reader agreement for the differentiation of benign and malignant lesions and for specific lesion diagnosis was determined using generalized kappa (κ) statistics. The agreement was classified as almost perfect (κ values > 0.8), substantial (κ = 0.61–0.8), moderate (κ = 0.41–0.6), fair (κ = 0.21–0.4) or none to slight (κ ≤ 0.2) [23]. Within-reader concordance (*i.e.*, the number of times complete agreement between HB-GBCAs was achieved) was determined both for malignant/benign lesion differentiation and for specific lesion diagnosis. Typical and atypical enhancement patterns of detected lesions were displayed descriptively, and inter-reader agreement was presented as percent agreement.

Finally, the confidence of each reader for lesion diagnosis was assessed using a derived 7-point scale that accounted for whether the readers assigned a single specific diagnosis for a given lesion or chose to assign differential diagnoses (*i.e*., 1, 2, or 3 differential diagnoses). The derived 7-point scale was established as follows: 7 = single diagnosis, correctly matched with final reference standard diagnosis; 6 = two differential diagnoses, the first correctly matched; 5 = two differential diagnoses, the second correctly matched; 4 = three differential diagnoses, the first correctly matched; 3 = three differential diagnoses, the second correctly matched; 2 = three differential diagnoses, the third correctly matched; 1 = no diagnostic match or lesions confirmed at final diagnosis but not detected at MRI. A comparison of mean confidence scores was performed using a paired *t-*test.

The study was powered to show a difference in accuracy of approximately 10% between HB-GBCAs for the differentiation of malignant and benign FLLs. Based on McNemar’s test of equality of paired proportions (nQuery, version 9.1.1; Statistical Solutions, Cork, Ireland), and assuming 24% discordant pairs, evaluation of 205 FLLs was necessary for 85% of power in a two-sided test with an alpha level of 0.05.

## Results

### Characteristics of patients and lesions

Overall, 140 patients (121 males, 86.4%, and 19 females, 13.6%; mean age ± standard deviation 52.3 ± 11.9 years) were included in the study. Ninety-two (65.7%) patients had known liver cirrhosis (Child A in 76/92 patients, 82.6%; Child B in 16/92 patients, 17.4%) while 48 (34.3%) patients had no diffuse or chronic liver disease.

A total of 208 lesions (mean size 43.7 ± 33.3 mm for malignant lesions and 18.15 ± 21.43 mm for benign lesions) were detected at final on-site diagnosis, comprising 96 HCC, 5 CC, 6 metastases, 1 bile cell carcinoma, 67 hemangiomas, 17 DNs, 9 focal nodular hyperplasias, 1 liver abscess, and 6 ‘other’ benign lesions (comprising 3 pseudolymphomas, 1 hamartoma and 2 inflammatory lesions). Of these 208 lesions, 126 were histologically confirmed via surgery and/or biopsy (all 108 malignant lesions and 18/100 benign lesions [9 hemangiomas, 2 DNs, 2 focal nodular hyperplasias, and 1 chronic inflammatory lesion at surgery; 3 DNs and 1 pseudolymphoma at biopsy]). The remaining 82 lesions were diagnosed as benign based on imaging follow-up, patient clinical history, and laboratory analysis.

### Lesion detection

Of the 208 lesions confirmed on-site, Readers 1, 2, and 3 failed to detect 32 (15.4%), 26 (12.5%), and 15 (7.2%) lesions, respectively, after gadobenate, and 32 (15.4%), 30 (14.4%), and 22 (10.6%) lesions, respectively, after gadoxetate (see Supplemental Table S1). Most missed lesions were hemangiomas (15, 7.2%; 22, 10.6%; and 11 5.3%) hemangiomas with both HB-GBCAs plus 3, 3, and 3 only with gadobenate and 5, 5, and 8 only with gadoxetate; readers 1, 2, and 3 respectively). All missed hemangiomas were < 1 cm in diameter apart from one hemangioma in the case of Reader 1. The second most frequent type of undetected lesion was DN: Readers 1, 2, and 3 missed 8 (3.8%), 8 (3.8%) and 6 (2.9%) DNs, respectively, after gadobenate, and 5 (2.4%), 5 (2.4%), and 7 (3.4%) DNs, respectively, after gadoxetate. All missed DNs were < 1.5 cm in diameter of which 6 were < 1 cm in diameter. In all but two cases the onsite final diagnosis for these lesions was imaging follow-up. Only three HCC nodules were < 1 cm in diameter. Readers 1, 2, and 3 missed all three of these lesions with gadoxetate, but only 2, 1, and 1 lesions, respectively, with gadobenate.

### Diagnostic performance

**Fig. 1:**
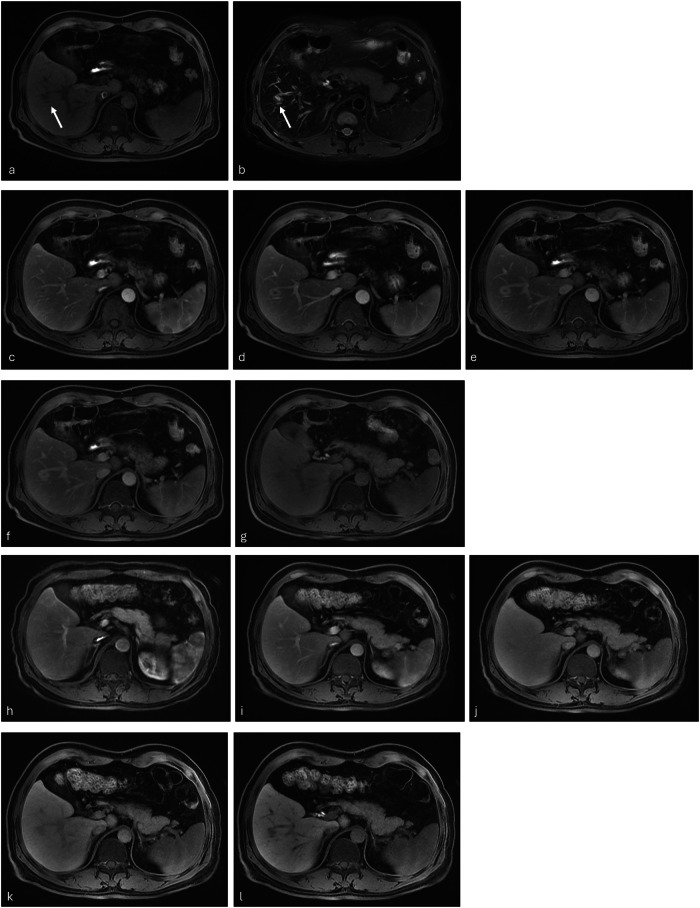
Small (1.5 cm) hepatic abscess. Unenhanced T1-weighted fat-saturated (**a**) and T2-weighted fat-saturated (**b**) images reveal a lesion that is hypointense and hyperintense, respectively, in the right lobe between segments V and VI (arrow). The lesion was diagnosed as benign (hemangioma) based on enhancement features on the late arterial (**c**), portal-venous (**d**), 3-min delayed (**e**), 5-min delayed (**f**), and HB (**g**) images after gadobenate administration. Conversely, it was diagnosed as malignant (metastasis) based on its appearance on the corresponding contrast-enhanced images (**h**–**l**) after gadoxetate administration. Histopathology confirmed a benign lesion (small hepatic abscess)

**Fig. 2:**
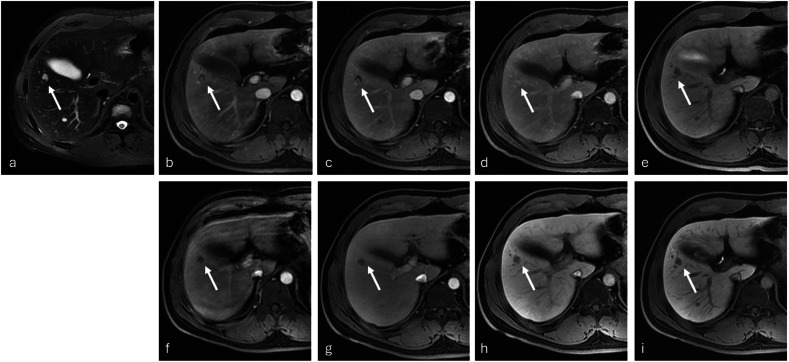
Small (1 cm) hemangioma. The unenhanced T2-weighted fat-saturated (**a**) image reveals a hyperintense lesion (arrow) in the right liver lobe, segment V. The lesion demonstrated enhancement features (“dot sign” enhancement and centripetal filling-in) characteristic of hemangioma on the late arterial (**b**), portal-venous (**c**) 5-min delayed (**d**) images after gadobenate administration followed by hypointensity in the HBP (**e**). Conversely, the lesion was hypointense during all corresponding dynamic phases (**f**–**h**, respectively) and the HBP (**i**) after gadoxetate administration leading to a diagnosis of metastasis. Note also the presence of motion artifacts on the early dynamic acquisition after gadoxetate administration. Imaging follow-up confirmed the diagnosis of a benign lesion, and imaging findings were consistent with hemangioma

Diagnostic performance for the differentiation of benign and malignant lesions is shown in Table 1. Significantly higher values for gadobenate were determined for all diagnostic performance parameters (Figs. 1 and 2) except for sensitivity (*p* = 0.083) and negative predictive value (*p* = 0.360) for Reader 1. Inter-reader agreement for malignant/benign lesion differentiation was markedly better with gadobenate (κ = 0.91) than with gadoxetate (κ = 0.72).

**Table 1:** Lesion level diagnostic performance for characterization of lesions as malignant or benign on dynamic and HB images enhanced with gadobenate and gadoxetate

Parameter	Reader	Gadobenate	Gadoxetate	*p*-value
Sensitivity*	1	91.6 (98/107)	88.8 (95/107)	0.083
	2	99.1 (106/107)	91.6 (98/107)	0.005
	3	94.4 (101/107)	86.0 (92/107)	0.007
Specificity*	1	87.5 (112/128)	83.6 (107/128)	0.025
	2	89.1 (114/128)	79.7 (102/128)	0.001
	3	90.5 (105/116)	83.6 (97/116)	0.011
Accuracy*	1	89.4 (210/235)	86.0 (202/235)	0.005
	2	93.6 (220/235)	85.1 (200/235)	< 0.001
	3	92.4 (206/223)	84.8 (189/223)	< 0.001
PPV**	1	86.0 (98/114)	78.0 (96/123)	< 0.001
	2	85.5 (106/124)	68.8 (99/144)	< 0.001
	3	89.4 (101/113)	76.2 (93/122)	< 0.001
NPV**	1	94.0 (140/149)	92.8 (155/167)	0.360
	2	99.3 (152/153)	93.8 (135/144)	< 0.001
	3	95.0 (113/119)	87.5 (105/120)	< 0.001

*NPV* Negative predictive value, *PPV* Positive predictive value

* McNemar’s test

** Wald test, adjusting within patient correlation due to multiple lesions from a patient

**Fig. 3:**
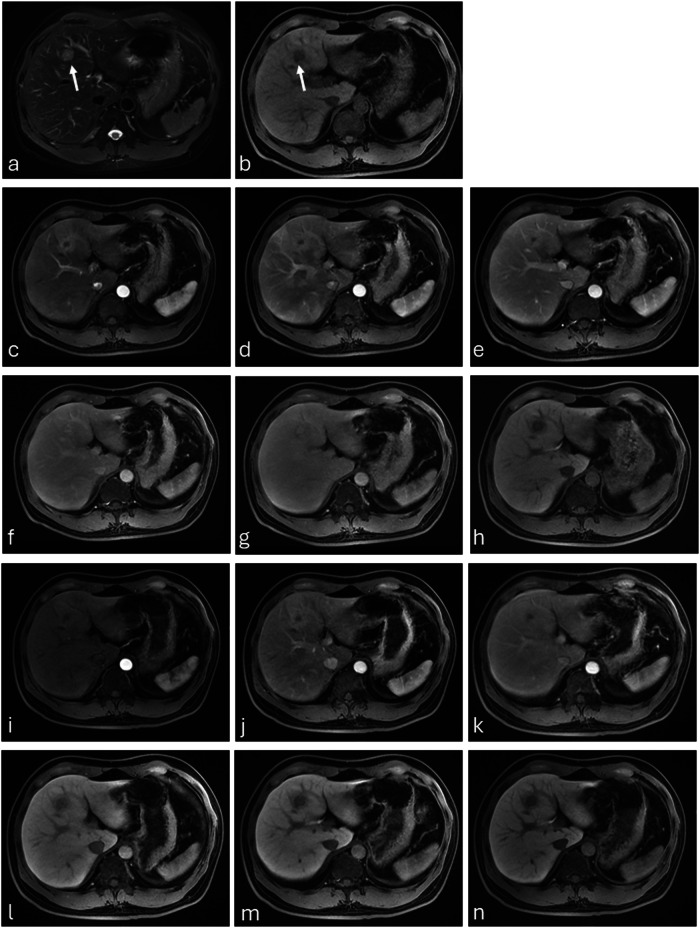
Cholangiocarcinoma (2 cm). Unenhanced T2-weighted fat-saturated (**a**) and T1-weighted fat-saturated (**b**) images reveal a lesion (arrow) that is hyperintense and hypointense, respectively, in segment IV of the left liver lobe. The lesion was diagnosed as cholangiocarcinoma based on enhancement features on the early arterial (**c**), late arterial (**d**), portal-venous (**e**), 5-min delayed (**f**), 10-min delayed (**g**), and HBP (**h**) images after gadobenate administration. Conversely, the lesion was diagnosed as a metastasis based on the corresponding images (**i**–**n**) acquired after gadoxetate administration. Histopathology confirmed a cholangiocarcinoma. Gadobenate-enhanced imaging allowed the detection of fibrotic tissue as hyperintensity during the delayed phase. A fibrotic component is a hallmark of cholangiocarcinoma

Readers 2 and 3 reported significantly greater accuracy for specific lesion diagnosis with gadobenate (Reader 2, 78.3% *versus* 69.6%, *p* < 0.001; Reader 3, 72.5% *versus* 62.8%, *p* < 0.001) (Table 2 and Fig. 3). Reader 1 also reported greater accuracy with gadobenate although the difference was not significant (72.0% *versus* 69.1%, *p* = 0.221). Inter-reader agreement for specific lesion diagnosis was moderate for both agents (κ = 0.49 and κ = 0.48 for gadobenate and gadoxetate, respectively).

**Table 2:** Accuracy for specific lesion diagnosis with gadobenate and gadoxetate

Reader	Gadobenate	Gadoxetate	*p-*value*
1	72.0 (149/207^a^)	69.1 (143/207)	0.221
2	78.3 (162/207)	69.6 (144/207)	< 0.001
3	72.5 (150/207)	62.8 (130/207)	< 0.001

* McNemar’s test

^a^ Includes all matched lesions on both dynamic and HBP images. One patient did not have HB images for gadobenate and was excluded from this analysis

**Fig. 4:**
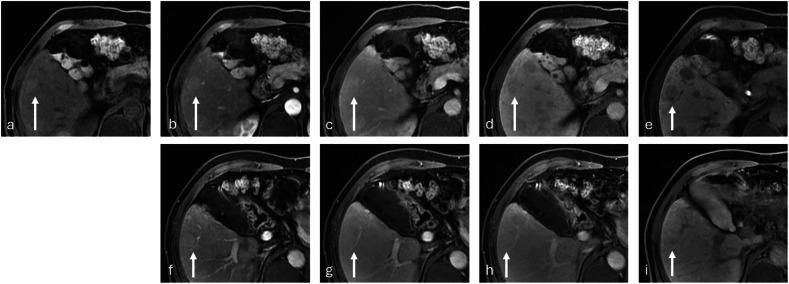
DN (2 cm). The unenhanced T1-weighted fat-saturated image (**a**) reveals a slightly hyperintense nodule (arrow) in segment V of the right liver lobe. After gadoxetate administration the lesion appeared hyperintense on the late arterial phase images (**b**), isointense on the portal-venous phase image (**c**), and hypointense on the 3-min delayed (**d**), and HBP (**e**) images. These features led to a diagnosis of early HCC. Conversely, the lesion demonstrated minimal hyperintensity in the late arterial phase (**f**), and isointensity in the portal-venous (**g**), 3-min delayed (**h**), and HBP (**i**) images after gadobenate administration. The lesion was diagnosed as a low-grade DN on gadobenate-enhanced imaging. Histopathology confirmed a low-grade DN

Among the 96 HCCs, the most frequent misdiagnosis was CC for Readers 1 and 2 (6/13 and 6/6 incorrect HCC diagnoses for gadobenate, respectively, and 5/17 and 10/13 incorrect HCC diagnoses for gadoxetate, respectively) and metastasis for Reader 3 (17/23 and 23/35 incorrect HCC diagnoses for gadobenate and gadoxetate, respectively) (see Supplemental Table S2). The most frequent benign (false negative) misdiagnosis of HCC was hemangioma (1 lesion misdiagnosed with gadobenate, and 2 lesions misdiagnosed with gadoxetate by Reader 1, and 3 lesions misdiagnosed with gadoxetate alone by Readers 2 and 3). When identified, the most challenging diagnosis was DN for which the rate of misdiagnosis was high, accounting for 7/9, 3/9, and 8/11 detected DN lesions for gadobenate and 8/12, 10/12, and 7/10 detected DN lesions for gadoxetate, for Readers 1, 2, and 3, respectively (Fig. 4).

Typical and atypical patterns of enhancement of individual FLLs seen by all three readers or by at least two readers (*n* = 172 lesions overall) are displayed in Table 3. All three readers agreed on the pattern of enhancement (either typical or atypical) for 144/172 (83.7%) lesions, with more “typical” enhancement patterns seen with gadobenate for HCC, CC, and hemangioma.

**Table 3:** Comparison of enhancement patterns of different FLLs with gadobenate and gadoxetate

Lesion type	Number of lesions*	Contrast agent	Typical pattern**	Atypical pattern**
HCC	95	Gadobenate	77	18
		Gadoxetate	65	30
CC	5	Gadobenate	5	0
		Gadoxetate	2	3
Metastasis	6	Gadobenate	3	3
		Gadoxetate	3	3
Hemangioma	48	Gadobenate	48	0
		Gadoxetate	37	11
Focal nodular hyperplasia	8	Gadobenate	7	1
		Gadoxetate	7	1
DN	10	Gadobenate	10	0
		Gadoxetate	10	0

* Number of lesions seen by all 3 readers or by at least 2/3 readers

** Typical patterns were defined as per criteria for FLL diagnosis from the AASLD [1]

The appearance of lesions in the HBP is shown in Table 4. Approximately 90% or more of all HCC nodules were hypointense in the HBP with both agents. Likewise, all metastases and most visualized hemangiomas were hypointense in the HBP with both agents.

**Table 4:** Appearance of lesions in the HBP

Number of lesions	Contrast agent	Appearance on HBP images														
		Hypointense			Isointense			Hyperintense			Targetoid			Not determined		
		R1	R2	R3	R1	R2	R3	R1	R2	R3	R1	R2	R3	R1	R2	R3
HCC (*n* = 96)	Gadobenate	87	90	86	4	4	4	1	1	5	2	0	0	2	1	1
	Gadoxetate	91	88	92	1	2	0	0	0	1	1	3	0	3	3	3
DN (*n* = 17)	Gadobenate	4	6	4	1	1	2	4	5	5	0	0	0	8	5	6
	Gadoxetate	5	6	5	1	1	1	6	5	5	0	0	0	5	5	6
CC (*n* = 5)	Gadobenate	3	4	4	0	1	0	0	0	1	2	0	0	0	0	0
	Gadoxetate	5	5	4	0	0	0	0	0	0	0	0	1	0	0	0
Metastases (*n* = 6)	Gadobenate	1	5	4	0	0	0	0	0	0	4	1	2	1	0	0
	Gadoxetate	5	6	5	0	0	0	0	0	0	0	0	0	1	0	1
Hemangioma (*n* = 67)	Gadobenate	44	39	51	4	3	1	1	0	1	0	0	0	18	25	14
	Gadoxetate	46	39	46	0	1	1	1	0	1	0	0	0	20	27	19
FNH (*n* = 8)	Gadobenate	3	2	2	2	5	2	3	1	4	0	0	0	0	0	0
	Gadoxetate	1	1	2	0	4	1	7	3	5	0	0	0	0	0	0

*CC* Cholangiocellular carcinoma, *DN* Dysplastic nodule, *FNH* Focal nodular hyperplasia, *HCC* Hepatocellular carcinoma, *R1* Reader 1, *R2* Reader 2, *R3* Reader 3

Within-reader concordance for the correct classification of lesion nature (malignant *versu* benign) between gadobenate and gadoxetate was high for all readers with slightly higher values for malignant lesions (88.9–92.6%) compared to benign lesions (78–84%) (Table 5). Likewise, within-reader concordance between gadobenate and gadoxetate for specific lesion diagnosis was high for all readers, again with slightly higher values for malignant lesions (83.3–89.8%) compared to benign lesions (78–81%) (Table 5).

**Table 5:** Concordance between gadobenate and gadoxetate for differentiation of malignant from benign lesions and specific lesion diagnosis

Reader	Concordance	Concordance for malignant/benign differentiation		Concordance for specific lesion diagnosis	
		Malignant (*n* = 108)	Benign (*n* = 100)	Malignant (*n* = 108)	Benign (*n* = 100)
1	Concordant	100 (92.6%)	80 (80.0%)	94 (87.0%)	79 (79.0%)
	Discordant	8 (7.4%)	20 (20.0%)	14 (13.0%)	21 (21.0%)
2	Concordant	100 (92.6%)	84 (84.0%)	97 (89.8%)	81 (81.0%)
	Discordant	8 (7.4%)	16 (16.0%)	11 (10.2%)	19 (19.0%)
3	Concordant	96 (88.9%)	78 (78.0%)	90 (83.3%)	78 (78.0%)
	Discordant	12 (11.1%)	22 (22.0%)	18 (16.7%)	23 (23.0%)

### Confidence in lesion diagnosis

For the confirmed lesions at final diagnosis, significantly higher mean derived confidence scores were obtained for gadobenate for Reader 2 (5.16 ± 2.71 *versus* 4.65 ± 2.93, *p* < 0.001) and Reader 3 (4.95 ± 2.79 *versus* 4.33 ± 2.97, *p* < 0.001), indicating more single diagnoses and fewer differential diagnoses with gadobenate. A higher mean-derived confidence score for gadobenate was also obtained for Reader 1, but the difference was not significant (5.08 ± 4.94 *versus* 4.94 ± 3.0, *p* = 0.341).

## Discussion

Our statistically powered, intra-individual crossover study demonstrates that diagnostic performance for the characterization of FLLs as malignant or benign is significantly better with gadobenate. All readers reported significantly higher values for sensitivity, specificity, accuracy, PPV, and NPV with gadobenate except for Reader 1 whose values for sensitivity and negative predictive value albeit higher with gadobenate, were not significantly higher. Likewise, the accuracy for specific lesion diagnosis was significantly greater with gadobenate for two of three blinded readers and non-significantly better with gadobenate for the remaining reader. Given that the appearance of lesions in the HBP was roughly similar for the two HB-GBCAs and that the within-reader concordance was high for both malignant and benign lesions, the better diagnostic performance with gadobenate can be ascribed in large part to its better dynamic phase imaging capability. As demonstrated previously, major LI-RADS features for the diagnosis of HCC, *i.e*., arterial phase hyperenhancement and wash-out on dynamic imaging, are more often seen with gadobenate than with gadoxetate [6, 7, 16]. The better dynamic imaging performance with gadobenate presumably also explains the better inter-reader agreement for malignant/benign lesion differentiation with gadobenate (κ = 0.91; almost perfect *versus* κ = 0.72; substantial, for gadoxetate) and the higher mean derived confidence scores for lesion diagnosis indicating more single diagnoses and fewer differential diagnoses with gadobenate.

The lesions most frequently missed by all three readers were small (typically < 1 cm) DNs and hemangiomas. While it is likely some very small lesions were missed because of partial volume phenomena, it should also be borne in mind that low-grade DN are sometimes problematic to detect and/or diagnose due to their enhancement features. Low-grade DNs are mainly supplied by the portal circulation and thus display enhancement characteristics similar to that of the background liver parenchyma on dynamic phase imaging [24]. Low-grade DNs also demonstrate organic anion transporting polypeptide 1B3 expression similar to or slightly higher than that of the surrounding liver parenchyma which frequently results in iso/hyperintensity relative to the surrounding liver in the HBP [24]. In our study, DN represented the most challenging diagnosis. The rate of misdiagnosis for lesions identified as DN was 7/9, 3/9, and 8/11 for gadobenate and 8/12, 10/12, and 7/10 for gadoxetate, for Readers 1, 2, and 3, respectively. In all but two cases the on-site final diagnosis of DN was based on imaging follow-up. The two histologically proven DN were classified as low-grade DN. These two lesions were slightly hypointense on the portal/TP with gadoxetate and were misinterpreted by all three readers as early HCC. The hypointensity of these lesions during the TP possibly reflects the fact that both the intracellular and extracellular pools of gadoxetate contribute substantially to parenchymal enhancement after the portal-venous phase resulting in a pseudo-washout appearance of certain lesions [25]. It is for this reason that TP images are not included in the LI-RADS lexicon for diagnosis of HCC on gadoxetate-enhanced images [25]. Although the lack of histological confirmation precludes an accurate explanation for the appearance of the other DNs, the three readers misdiagnosed these lesions as early HCC in most cases on images acquired with gadoxetate. Based on the latest guidelines [26, 27], DNs should not be treated or managed as cancers, and low-grade DNs should be followed up by regular imaging studies.

Concerning other misdiagnoses, it is noteworthy that Reader 2 misinterpreted 3 of 6 metastatic lesions as benign (hemangiomas) with gadoxetate, while misinterpreting 4 of these 6 lesions as still malignant (1 HCC; 3 CC) with gadobenate. No metastatic lesions were misinterpreted as benign by Readers 1 and 3 either with gadobenate or gadoxetate. Previous studies on gadoxetate-enhanced MRI have shown that hemangiomas, particularly small hemangiomas, frequently have decreased signal intensity during the portal-venous phase which may mimic metastasis [5, 28]. Again, this is due to a pseudo-washout appearance on late dynamic acquisitions [29] which does not occur with gadobenate [12]. Although bright signal intensity on T2-weighted images is considered a key feature suggestive of hemangioma, this can sometimes be missed in small lesions because of partial volume phenomena [29]. Notably, hemangioma was the most frequent benign misdiagnosis for HCC in our study, with Reader 1 misdiagnosing 1 lesion with gadobenate and 2 lesions with gadoxetate, and Readers 2 and 3 misdiagnosing 3 lesions, all with gadoxetate. Hemangiomas with diffuse enhancement during the arterial phase frequently show pseudo-washout during late-phase gadoxetate-enhanced MRI, making their differentiation from hypervascular malignancies like HCC difficult [12, 29]. Successful differentiation of hemangiomas from malignant FLLs is clinically very important because the former usually require no treatment. In our study, all 48 hemangiomas seen by at least two readers showed a typical enhancement pattern with gadobenate, compared with only 37 hemangiomas with gadoxetate.

Regarding CC, delayed persistent enhancement of the tumor nodule was identified in the late dynamic phase with gadobenate. This finding reflects the abundant fibrous content of the tumor and the slow diffusion of contrast agents into the tumor interstitium [30]. On the other hand, with gadoxetate, this delayed enhancement was not always detected due to early hepatocyte-specific enhancement of the liver parenchyma. Notably, in 2 of the 5 patients with CC who had Child A cirrhosis, the CC nodules were hypervascular during the arterial phase mimicking HCC [31].

The addition of clinical information is known to significantly improve the diagnostic performance of liver MRI [32]. In our study, an assessment of images was performed in the absence of any patient clinical or radiological information. Given these circumstances, our sensitivity and specificity findings of above 90% and 85% for gadobenate for the differentiation of benign from malignant FLL can be considered remarkable. It is likely that improved results would have been achieved had clinical and radiological information been available at the time of image evaluation.

This study has some limitations. First, while prospective in design with adequate statistical power, the study did not include a wide variety of FLLs. The majority of lesions were primarily HCC and hemangioma. Further studies with a larger representation of focal nodular hyperplasia, CC, metastases, and adenomas may be warranted to confirm our findings and further support the study’s conclusions. Second, the study was conducted at a single center in China using a 3-T magnet with patient clinical characteristics typical of the Asian population. In this regard, the imbalance between cirrhotic (*n* = 92) and non-cirrhotic (*n* = 48) patients might have affected the results. Since cirrhosis can be recognized merely by looking at the images, this may have impacted the expectation of certain types of lesions in a cirrhotic patient (*e.g*., DN, regenerating nodule, HCC) compared to a patient with no diffuse liver disease. Third, while all malignant lesions were histologically confirmed, most benign lesions lacked pathological confirmation. Fourth, a standard injection rate of 2 mL/s was used for both gadobenate and gadoxetate. Previous studies have suggested that a slower injection rate of 1 mL/s may avoid the abrupt concentration changes during k-space acquisition leading to a reduction of truncation artifacts during the arterial phase for gadoxetate [33, 34]. Finally, a blinded assessment of images considered the complete MRI examination comprising unenhanced images and enhanced dynamic and HBP images combined. However, many centers acquire only the pre-contrast plus contrast-enhanced dynamic phase images when using gadobenate. Previous studies have confirmed the additional contribution that HBP images can make over and beyond dynamic phase images alone [19, 35, 36].

In conclusion, this prospective crossover study confirmed that gadobenate is superior to gadoxetate for the differentiation of benign and malignant lesions, providing significantly better sensitivity and specificity for lesion differentiation and more accurate specific lesion diagnosis when the entire MRI examination, including unenhanced, dynamic, and HBP images, are considered. Further confirmatory studies that include a wider representation of different types of FLL are warranted.

## Supplementary information

**Additional file 1:** **Supplemental Table 1.** Lesions undetected by Readers 1, 2, and 3. **Supplemental Table 2.** Number and type of misdiagnosis for the most represented FLLs.

## Data Availability

All data generated or analyzed during this study are included in this published article.

## Abbreviations

CC: Cholangiocellular carcinoma
DN: Dysplastic nodule
FLL: Focal liver lesion
GBCA: Gadolinium-based contrast agent
HB: Hepatobiliary
HBP: Hepatobiliary phase
HCC: Hepatocellular carcinoma
LI-RADS: Liver imaging reporting and data system
MRI: Magnetic resonance imaging
TP: Transitional phase
