# Supplementary material for: Intraindividual crossover comparison of gadobenate dimeglumine-enhanced and gadoxetate disodium-enhanced MRI for characterizing focal liver lesions
Source: Eur Radiol Exp. 2025 Feb 18;9:23. doi: 10.1186/s41747-025-00551-8 (PMC11836252; doi:10.1186/s41747-025-00551-8)
Supplement: Supplementary file 1 — Additional file 1: Supplemental Table 1. Lesions undetected by Readers 1, 2, and 3. Supplemental Table 2. Number and type of misdiagnosis for the most represented FLLs. [file 41747_2025_551_MOESM1_ESM.pdf]

Intraindividual crossover comparison of gadobenate dimeglumine-enhanced and gadoxetate disodium-enhanced MRI for characterizing focal liver lesions

ELECTRONIC SUPPLEMENTARY MATERIAL

Supplemental Table 1. Lesions undetected by Readers 1, 2, and 3

Reader and agent	HCC	Metastasis	Hemangioma	Dysplastic Nodule	Other benign	Other benign	Other benign	Total
READER 1								
Only gadobenate	0	1	3	0	1	1	1	15
Only gadoxetate	1	1	5	0	1	1	1	15
Both agents	2	0	15	0	0	0	0	17
Total gadobenate	2	1	18	8	1	1	1	32
Total gadoxetate	3	1	20	5	1	1	1	32
READER 2								
Only gadobenate	0	0	3	0	0	0	0	3
Only gadoxetate	2	0	5	0	0	0	0	7
Both agents	1	0	22	0	0	0	0	23
Total gadobenate	1	0	25	8	0	0	0	26
Total gadoxetate	3	0	27	5	0	0	0	30
READER 3								
Only gadobenate	0	0	3	0	0	0	0	3
Only gadoxetate	2	0	8	0	0	0	0	10
Both agents	1	0	11	0	0	0	0	12
Total gadobenate	1	0	14	6	0	0	0	15
Total gadoxetate	3	0	19	7	0	0	0	22

Supplemental Table 2. Number and type of misdiagnosis for the most represented FLLs

Type of lesion	Reader	Agent	Undetected	Correct diagnoses	Incorrect diagnoses	Type of incorrect diagnoses
HCC = 96	1	Gadobenate	2	81	13	CC = 6; Metastasis = 1; FNH = 1; HA = 3; DN = 1; HEM =1
		Gadoxetate	3	76	17	CC = 5; Metastasis = 4; Gallbladder carcinoma = 1; HA = 3; DN = 1; HEM = 2; Hamartoma = 1
	2	Gadobenate	1	89	6	CC = 6
		Gadoxetate	3	80	13	CC = 10; HEM =3
	3	Gadobenate	1	72	23	CC = 1; Metastasis = 17; HA = 5
		Gadoxetate	3	58	35	CC = 1; Metastasis = 23; HA = 8; DN = 1; HEM =2
CC = 5	1	Gadobenate	0	4	1	HCC = 1
		Gadoxetate	0	3	2	HCC = 1; Abscess = 1
	2	Gadobenate	0	4	1	HCC = 1
		Gadoxetate	0	3	2	HCC = 2
	3	Gadobenate	0	3	2	Metastasis = 2
		Gadoxetate	0	2	3	Metastasis = 3
Metastasis = 6	1	Gadobenate	1	4	1	CC = 1
		Gadoxetate	1	4	1	CC = 1
	2	Gadobenate	0	2	4	HCC = 1; CC = 3
		Gadoxetate	0	0	6	CC = 3; HEM = 3
	3	Gadobenate	0	6	0	
		Gadoxetate	1	4	1	HCC = 1
DN = 17	1	Gadobenate	8	2	7	HCC = 2; HA = 3; RN = 2
		Gadoxetate	5	4	8	HCC = 6; FNH = 2
	2	Gadobenate	8	6	3	HCC = 3
		Gadoxetate	5	2	10	HCC = 7; FNH = 3
	3	Gadobenate	6	3	8	HCC = 2; HA = 5; OTHER = 1
		Gadoxetate	7	3	7	HCC = 4; FNH = 1; HA = 2
HEM = 67	1	Gadobenate	18	47	2	FNH = 2
		Gadoxetate	20	45	2	FNH = 1; Pseudolesion = 1

Type of lesion	Reader	Agent	Undetected	Correct diagnoses	Incorrect diagnoses	Type of incorrect diagnoses
	2	Gadobenate	25	40	2	FNH = 2
		Gadoxetate	27	38	2	HCC = 1; FNH = 1
	3	Gadobenate	14	49	4	FNH = 2; HEM = 2
		Gadoxetate	19	44	4	HCC = 1; FNH = 1; HEM = 2
FNH = 8	1	Gadobenate	0	7	1	HA = 1
		Gadoxetate	0	7	1	HA = 1
	2	Gadobenate	0	8	0	
		Gadoxetate	0	7	1	HCC = 1
	3	Gadobenate	0	8	0	
		Gadoxetate	0	6	2	Metastasis = 1; HA = 1
HCC = Hepatocellular carcinoma; CC = Cholangiocellular carcinoma; DN = Dysplastic Nodule; RN = Regenerating Nodule; HEM = Hemangioma; HA = Hepatic Adenoma; FNH = Focal Nodular Hyperplasia						
